# Lack of uniformity in screening, diagnosis and management of gestational diabetes mellitus among health practitioners across major cities of Pakistan

**DOI:** 10.12669/pjms.342.12213

**Published:** 2018

**Authors:** Syed Hunain Riaz, Muhammad Shais Khan, Ali Jawa, Madeeha Hassan, Javed Akram

**Affiliations:** 1 Dr. Syed Hunain Riaz. FCPS. Wilshire Cardiovascular & Endocrine Center of Excellence (WILCARE), Lahore, Pakistan; 2 Dr.Muhammad Shais Khan, Shaheed Zulfiqar Ali Bhutto Medical University, PIMS, Islamabad, Pakistan. Federal Medical & Dental College, Islamabad, Pakistan; 3 Prof. Ali Jawa, MD, MPH, FACE, DABIM, DABPNS, FRCP, MIVMC Shaheed Zulfiqar Ali Bhutto Medical University, PIMS, Islamabad, Pakistan. Federal Medical & Dental College, Islamabad, Pakistan; 4 Dr.Madeeha Hassan. Shaheed Zulfiqar Ali Bhutto Medical University, PIMS, Islamabad, Pakistan. Federal Medical & Dental College, Islamabad, Pakistan; 5 Prof. Javed Akram. MD, MRCP, FRCP, FRCP, FACP, FACC, FASIM. Shaheed Zulfiqar Ali Bhutto Medical University, PIMS, Islamabad, Pakistan. Federal Medical & Dental College, Islamabad, Pakistan

**Keywords:** Gestational diabetes mellitus, GDM Diagnosis, Oral glucose tolerance test, GDM treatment, Pakistan, South Asian federation of Endocrine societies

## Abstract

**Objective:**

To determine knowledge, attitude and practice (KAP) regarding management of Gestational Diabetes Mellitus (GDM) among Health Care Providers in major cities of Pakistan.

**Methods:**

A knowledge, attitude and practice (KAP) questionnaire based study was conducted in major cities in Pakistan from health care providers in public and private hospitals and clinics. Questionnaires were provided to the health care providers regarding screening, diagnosis and management of patients with GDM. Data analysis was done using IBM SPSS 20.

**Results:**

A total of 210 doctors took part in the study. 55 (26%) reported using fasting blood glucose as screening test for GDM whereas 129(61.4%) respondents used Oral Glucose Tolerance based WHO criteria for diagnosing GDM. Thirty six (17%) and 98(46.7%) doctors referred their patients to Gynecologists. For treating GDM, 64(30.5%) doctors prescribed insulin (NPH/Regular, 70/30 Mix). 112 (53.5) doctors used combination of capillary glucose by glucometer and plasma blood glucose tests for monitoring of glycemic control of patients with GDM.

**Conclusion:**

There is lack of agreed screening tests and criteria for diagnosis and management of GDM patients. Doctors need to be educated to follow evidence based diagnostic and management guidelines so that GDM patients can be effectively managed. Recently released South Asian Federation Societies and Pakistan Endocrine Society guidelines could be much needed consensus guidelines for doctors to apply in their daily practice to improve GDM diagnosis and treatment.

## INTRODUCTION

Gestational diabetes mellitus (GDM) is an endocrine disorder resulting in hyperglycemia with first recognition during pregnancy affecting a significant number of pregnancies each year.1 Associated with high risk of perinatal complications, GDM is the most common medical condition in pregnancy affecting 1-14% of pregnancies. Maternal and fetal complications associated with GDM are largely preventable with early recognition and treatment.^2^ In middle to low income countries like Pakistan, high maternal and neonatal mortality and morbidity underscores the importance of early recognition and treatment of GDM by health care providers.

Globally there is a lack of consensus regarding diagnosis and further management of GDM and different approaches are adhered to in different region of the world due to lack of a universally accepted protocol. Different protocols used internationally recommend their own patient selection criteria and diagnostic thresholds. Currently the protocols used for diagnosis follow either the 75gm or the 100gm oral glucose tolerance tests (OGTT).^3^ The 75gm OGTT guidance by the International Association of Diabetes and Pregnancy Study Groups (IADPSG) recommends testing between 24-28 weeks of gestation in all women who have not been diagnosed with DM during the first ante-natal visit The diagnosis is established with at least two out of three elevated plasma glucose readings as follows: fasting>92mg/dl,1-hour post 75 glucose ingestion>180mg/dl, and 2-hour post ingestion>153mg/dl.^4^ The American Diabetes Association (ADA) recommendation glycemic thresholds with 75gm OGTT are also similar to the IADPSG.^5^ The WHO criteria (75gm OGTT) glycemic thresholds for diagnosis of GDM are the same as for a people with impaired glucose tolerance (IGT) and DM outside pregnancy.^6^

Despite considerable advances in medical approach there have been considerable gaps between disease outcome and acceptable treatment.^7^,^8^ The purpose of this GDM KAP study was to investigate knowledge & attitude of health care providers regarding GDM and to identify differences in the practice and approach among them.

## METHODS

A nation-wide questionnaire based survey was conducted to study practices regarding screening, diagnosis, treatment and follow-up of GDM in health care practitioners across 21 major cities of Pakistan. Data was collected on a structured self-reported standardized multiple response questionnaire. The questionnaire assessed the knowledge and current practices of health care professionals across following domains of GDM: Timing of screening with respect to trimester of pregnancy, methods and guidelines used for screening and diagnosis of GDM, treatment strategies used for management of GDM and follow-up plan for detection of persistent DM or new onset T2DM.

Volunteer medical students from Federal Medical and Dental Medical College, Islamabad, belonging to diverse cities of Pakistan were enrolled in the study. The questionnaire proforma was explained by the lead investigator and the proformas handed over to them just before college summer break. Questionnaires were then taken by them to the practitioners to be filled on the spot and returned. Four hundred (400) health care practitioners including gynecologists, endocrinologists, medical specialists, family physicians and medical officers were contacted. Data was analyzed using IBM SPSS 20. Ethical approval for the study was provided by Ethics Review Board of Shaheed Zulfiqar Ali Bhutto Medical University, PIMS, Islamabad, Pakistan.

## RESULTS

Two hundred and ten health care practitioners out of 400 hundred contacted from a number of major cities of Pakistan (Table-I) took part in the research. The response rate was 52.5%. Respondents included 107 (50.95%) gynecologists, 69 (32.9%) family physicians, 3 (1.4%) endocrinologists, 23 (10.9%) consultant physicians and 8 (3.8%) medical officers. Health care practitioners from public and private sectors were included in the study.

**Table-I:** Geographical distribution of respondents.

Major Cities of Pakistan	Respondents Percentage
Gilgit	1%
Skardu	1.4%
Islamabad	24.8%
Wah Cant.	1%
Taxilla	2.9%
Hasan Abdal	0.5%
Peshawar	5.2%
Rawalpindi	11.9%
Shakargarh	0.5%
Sialkot	4.8%
Sarghodha	1.4%
Faisalabad	4.3%
Gujranwala	4.8%
Lahore	5.7%
Toba Tek Singh	1%
Gojra	1%
Multan	4.8%
Quetta	2.4%
Mirpurkhas	4.8%
Hyderabad	4.8%
Karachi	11.4%

Two hundred and two (96.2%) were seeing patients already diagnosed with GDM and 207 (98.6%) were screening their patients for GDM. A large number of respondents (n=82 [39%]) were screening patients for GDM during first presentation to antenatal clinics regardless of timing of pregnancy. Eighty-nine (42.4%) respondents were screening for GDM specifically during 2^nd^ and 3^rd^ trimester of pregnancy. Rest of the respondents (n=39 [18.6%]) said that they were screening their patients during routine clinical visits. Although most of the respondents (62.4%) were screening all antenatal patients for GDM, a large number of respondents only screened patients if they either had signs and symptoms suggestive of GDM (n=17 [8.1%]) or were at high risk for GDM (n=51 [24.3%]). Eleven respondents (5.2%) were not screening patients for GDM at all. Most of the respondents (n=90 [42.9%]) were seeing less than five patients of GDM each month. Seventy respondents (33.3%) were seeing between 6 and10 patients, 23(11%) between 11 and 20 patients and 27 (12.9%) more than 20 patients of GDM per month. Only 61 (29.1%) practitioners were using an oral glucose tolerance test (OGTT) for screening purposes; these included 50 gram OGTT by 9 (4.29%), 75 gram OGTT by 51 (24.29%) and 100 gram OGTT by 1 (0.5%) respondent. A venous blood fasting glucose was being used as screening test by 55 (26.19%) respondents. Other screening tests being used included: a random venous blood glucose by 53 (25.24%), a fasting capillary blood glucose using glucometer by 5 (2.85%), a random capillary blood glucose using glucometer by 8 (3.81%) and HbA1c by 20 (9.52%) respondents (Table-II).

**Table-II:** Screening method used by respondents.

Screening method	Percentage
Fasting blood glucose (lab)	26.19%
Random blood glucose (lab)	25.24%
75 gm OGTT	24.29%
HbA1C	9.52%
50 gm OGTT	4.29%
Random capillary glucose (Glucometer)	3.81%
Fasting capillary glucose (Glucometer)	2.85%
Urine complete examination	3.81%

There were no uniform criteria used for the diagnosis of GDM. Most of the respondents (n= 129 [61.4%]) reported to be following WHO criteria for the diagnosis of GDM. Other criteria used included ADA guidelines by 26 (12.4%) and IADPSG (International Association of Diabetes and Pregnancy Study Groups) criteria by 19 (9%) respondents. Thirty-six (17.1%) practitioners were not sure about the criteria they were using (Table-III). The reported approximate frequency of GDM among screened cases by practitioners had a wide range i.e. less than 1% by 34 (16.2%), 1-5% by 69 (32.9%), 5-10% by 61 (29%), 10-20% by 22 (10.5%) and as high as 20-30% by 34 (11.4%) respondents. Most practitioners (n=117 [55.7%]) were managing GDM on their own while 93 (44.3%) referred their patients. Most common regimens prescribed for treatment of GDM included insulin (NPH/Regular 70/30) by 64 (30.5%), insulin (NPH/Regular in split regimes) by 60 (28.6%), a combination of insulin and oral anti-diabetic drugs by 35 (16.7%), oral anti-diabetic agents by 37 (17.6%), basal insulin only by 11 (5.2%) and basal plus ultra-short acting insulin by 3 (1.4%) respondents.(Table-IV)

**Table-III:** Criteria for oral glucose tolerance testing in GDM used by respondents.

Criteria	Percentage
WHO	61.43%
Not sure	17.14%
ADA	12.38%
IADPS	9.05%

Fifty seven (27.1%) respondents were following up their patients by capillary glucose monitoring using glucometer, 41(19.5%) by plasma blood glucose from lab and 112(53.5%) by a combination of both. A significant number of respondents were not aware of right timing of postpartum evaluation for persistent diabetes. Forty one (19.5%) respondents had been asking the patients to visit next day; 58(27.6%) were screening after one month and 111(52.9%) after six weeks of delivery. Investigations done for follow up of persistent diabetes cases by 75(35.7%) was fasting blood sugar, 32(15.2%) by random blood sugar from lab, 6(2.9%) by fasting blood sugar by glucometer, 17(8.1%) by random blood sugar levels (glucometer), 41(19.5%) by HbA1c, 12(5.7%) by 100 gram OGTT, 24 (11.4%) by 75 gram OGTT, 2(1%) by 50 gram OGTT and 1(0.5%) urine for glycosuria.

**Table-IV:** Treatment prescribed by respondents.

Treatment	Percentage
Insulin 70/30	30.48%
Insulin split regimen	28.57%
Oral Hypoglycemics	17.62%
Insulin + Oral Hypoglycemics	16.67%
Basal Insulin	5.24%
Basal Insulin + Ultra short Insulin	1.43%

**Table-V:** Follow up investigations used by respondents for persistent diabetes.

Investigation	Percentage
Fasting blood glucose (lab)	35.71%
HbA1C	19.52
Random blood glucose (lab)	15.24%
75 gm OGTT	11.43%
Random capillary glucose(glucometer)	8.10%
100 gm OGTT	5.71%
Fasting capillary glucose(glucometer)	2.86%
50 gm OGTT	0.95%
Urine c/e	0.48%

If the initial postpartum follow-up evaluation was negative for persistent diabetes, 103 (49.5%) doctors were screening patients for development of T2DM after 6 months, 35 (16.7%) after one year, 12 (5.7%) after three years. A large number (n=60 [28.6%]) were not screening patients for T2DM at all.

## DISCUSSION

Our study shows there is clear lack of consensus among health care providers regarding management of GDM. According to our study GDM was mostly managed by gynecologists (51%). Screening was mostly done on first presentation of patient to antenatal clinic as compared to widely adopted timing that is 2^nd^ trimester.4 Timing of screening is important especially in cases of patients who are at higher risk for preexisting DM or GDM. 39% in our study screened patients during first antenatal visit regardless of timing of visit 62% screen all antenatal patients whereas 24% screen only high risk patients. Physicians screening their patients only on the basis of risk factors might miss at least 30% of the cases which may lead to serious pregnancy outcomes.^4^ A KAP study by Divakar showed that 65% screened for GDM using blood glucose during first antenatal visit.^15^ In our study OGTT was the most common screening test (29%) out of which the 75gm version was the most used (24%). Fasting plasma glucose was used to screen in 26%. A retrospective study by J.Akhtar et al in 1996 showed that the GCT/OGTT (75 gm) screening test was the most used in a tertiary care hospital.^9^ Randhawa et al in 2003 also used GCT/OGTT(75gm) for screening in their prospective study.^10^ A study from conducted in Punjab, India by Gupta et al showed that only 3.8% doctors relied on OGTT to screen while 78% relied on fasting blood glucose for screening and diagnosis.^11^ A KAP study from India showed 96% doctors using OGTT to screen but only 42% of them actually knowing the cut off values for diagnosis.^12^ The aforementioned shows that there is a similar pattern of non-homogeneity in management protocol of GDM in the region. Even though Europe has a prevalence of GDM of 2-6% only but still there is a lack consensus regarding screening tests and glycemic thresholds and timing of screening. A review article from Europe quoted the use of OGTT (50 or 100 gm) with or without GCT.^13^

In the treatment aspect, again a lack of agreement was seen among the doctors in our study. Most of doctors, about 59% prescribe insulin in either NPH/Regular 70/30 fixed regimen or NPH/Regular split regimen. Gupta et al showed that 79% doctors preferred insulin as the treatment of choice.^11^ A review article showed that evidence suggests that metformin has proved to be a very effective option in providing good blood sugar control, good safety and outcomes are improved when Insulin is supplemented with metformin.^13^ However health care providers in our setting appear to be reluctant in using metformin as an alternate to insulin reflecting an apparent gap in up to date knowledge regarding GDM management. Insulin is the recommended first line treatment for GDM in the U.S followed by metformin.^14^

During the follow up of GDM patients, in our study 27% preferred glucometer readings while 20% opted for venous blood glucose readings from lab. Divakar showed that 41% clinicians preferred venous blood glucose levels at two weekly intervals and did not recommend home monitoring with glucometer.^15^ In our study, patients were asked six weeks after delivery to follow up by 53% while Babu et al showed that up to 85% clinicians in public health facilities recommended follow up at 6 weeks.^12^ This is a contrasting difference in practice across the region.

On follow up post-partum, in our study, again a mixed practice was seen. Fasting blood sugar from lab was the most common investigation, ordered by 36% and OGTT with 75 gm glucose by 12% while an Indian study showed 17% of clinicians recommended 75 gm OGTT at 6 weeks post-partum. The American Diabetes Association (ADA) recommends post-partum follow up at 6-12 weeks and testing with either fasting venous blood glucose or 75 gm OGTT.^5^ If the results of the post-partum screening were found to be normal then 50% of doctors in our study screened again at six months while 6% screened after three years and alarmingly 29% didn’t screen at all. This 29% reflects a hiatus in updated management awareness among the health care providers as ADA recommends that if post-partum screening is negative then retesting should be done in three years’ time and if early screening revealed impaired fasting glucose or impaired glucose tolerance then annual screening is recommended.

### Limitation

As ours is a KAP study and we don’t have actual patient data and available data is based on what the doctors can recall so chances of bias cannot be ruled out.

## CONCLUSION

Gestational diabetes mellitus is a high-risk state with increased morbidity and mortality in both mother and fetus. Proper knowledge, attitude and practices of health care providers in this regard are needed for early detection and intervention for good pregnancy outcome. There is a lack of agreed screening tests and criteria for diagnosis and management of GDM and health care providers are not abreast with the latest evidence based recommendations internationally. Both maternal and fetal mortality and morbidity can be prevented by establishing well-defined universal criteria to screen, treat, manage and follow-up a patient with GDM and educate and update health care providers concerned with GDM management with the latest practices. The recently launched South Asian Federation of Endocrine Societies Guidelines are indeed very helpful. It is imperative that the GDM guidelines are aggressively propagated among Gynecologists and General Physicians so that diagnosis and treatment are optimized.
